# Neuroimmune crosstalk in cancer: emerging immunotherapeutic opportunities

**DOI:** 10.1136/jitc-2026-015675

**Published:** 2026-10-21

**Authors:** Tianhui Dong, Hidde Ploeger, Tuany Eichwald, Laura Brabenec, Moutih Rafei, Moran Amit, Alessandra Gargano, Sebastien Talbot

**Affiliations:** 1 Department of Biomedical and Molecular Sciences, Queen’s University, Kingston, Ontario, Canada; 2 TRON-Translational Oncology at the University Medical Cente, Johannes Gutenberg University Mainz, Mainz, Germany; 3 Department of Physiology and Pharmacology, Karolinska Institutet, Stockholm, Sweden; 4 Département de Pharmacologie et Physiologie, Université de Montréal, Montreal, Québec, Canada; 5 Department of Head and Neck Surgery, The University of Texas MD Anderson Cancer Center, Houston, Texas, USA; 6 TRON-Translational Oncology, Johannes Gutenberg University Mainz, Mainz, Germany

**Keywords:** Tumor microenvironment - TME, Neuropathy, Immune Checkpoint Inhibitor, Immunotherapy, Adoptive cell therapy - ACT

## Abstract

The nervous system is increasingly recognized as an active regulatory layer of the tumor microenvironment. Tumors remodel local innervation, engage peripheral and central neural circuits, and mobilize neurotransmitters, neuropeptides, neurotrophic cues, and systemic neuroendocrine outputs that influence growth, invasion, immune evasion, therapeutic response, and toxicity. Tumor and stromal cells can also adopt neural-like phenotypes, extending tumor-neural communication beyond anatomically defined nerves. A unifying model is emerging: homeostatic neural inputs may support tissue integrity and immune surveillance, whereas tumor-activated or tumor-injured pathways can promote immune escape, dissemination, and immunotherapy resistance. This review follows that progression from tumor-induced neural remodeling and neural-like cell states to the sensory, autonomic, enteric, and systemic neuroendocrine axes that regulate antitumor immunity. We then consider how spatial omics resolves these interactions in situ and how neuroimmune pathways may be targeted to improve immunotherapy efficacy while limiting immune-related adverse events. Together, these advances position cancer neuroimmunology as a framework for interpreting tumor ecosystems and developing neuro-informed immunotherapies.

## Introduction

 The nervous system is now recognized as an active regulatory compartment of the tumor microenvironment (TME), not merely a structural scaffold. Neural elements communicate bidirectionally with malignant, stromal, vascular, and immune cells. Tumors can induce axonogenesis, alter neuronal excitability, and signal to local and systemic circuits through neurotrophic factors, cytokines, and axon-guidance cues.^1^ Nerves, in turn, release neurotransmitters and neuropeptides that regulate cancer-cell behavior, vascular remodeling, and antitumor immunity, creating a tumor-neural ecosystem that shapes progression and treatment response.^2 3^

Neural programs that normally coordinate tissue repair, inflammatory restraint, stress adaptation, and disease tolerance can preserve homeostasis during acute injury. In cancer, persistent inflammation, neural activation, and nerve injury can redirect the same programs toward immune exclusion or tolerance. This distinction explains why intact neural inputs may support immune surveillance in some settings, whereas tumor-activated or injured pathways can promote invasion, immunosuppression, and therapy resistance.^4–6^ A second layer of complexity comes from the tumor cells themselves. Neural features are not confined to cancers of bona fide neural origin: some malignancies retain lineage-derived neural properties, whereas others acquire neural-like transcriptional and functional programs through adaptive reprogramming. Distinguishing retained lineage identity from inducible neural mimicry is therefore essential for interpreting tumor-neural interactions and for identifying mechanisms shared across cancer types.

The review is organized around this movement from structure to mechanism to therapy. We first discuss tumor-induced neural remodeling and neural-like tumor states, then map the sensory, autonomic, enteric, and systemic neuroendocrine axes that shape antitumor immunity. We next show how spatial omics places these interactions within intact tissue architecture, before turning to implications for immune checkpoint blockade, adoptive cell therapies, and immunotherapy-related adverse events.

## Tumor-induced neural remodeling and activation

The argument begins with anatomy, because tumors can remodel and activate nearby nerves in both brain and peripheral malignancies.^7–9^ Tumor cells engage adjacent nerves through neurotrophic factors, cytokines, and axon-guidance molecules that promote nerve sprouting, structural remodeling, and increased neuronal activity.^10 11^ Once activated, nerves release neurotransmitters such as acetylcholine, glutamate, and serotonin, as well as neuropeptides including substance P, calcitonin gene-related peptide (CGRP), neuropeptide Y, and galanin, which can support tumor-cell proliferation, migration, angiogenesis, and immune modulation.^5 12–15^ These reciprocal interactions create a tumor-supportive neural niche in which nerve density, nerve activity, and immune organization are coupled to invasion and disease progression.^16^

These anatomical changes are driven by convergent molecular programs. Among the best characterized are the neurotrophins nerve growth factor (NGF), brain-derived neurotrophic factor (BDNF), and glial cell line-derived neurotrophic factor (GDNF). NGF signals through tropomyosin receptor kinase A (TrkA) and nerve growth factor receptor (NGFR; also known as p75 neurotrophin receptor, p75NTR), whereas pro-NGF preferentially signals through an NGFR/p75NTR-sortilin complex. In breast cancer, tumor-derived NGF promotes angiogenesis, axonal growth, and downstream CGRP expression.^17–20^ BDNF and GDNF show how neurotrophic remodeling becomes tissue-specific. In glioma, BDNF is upregulated and has been explored as a biomarker^21^; at glioma-neuron synapses, BDNF-TrkB signaling enhances α-amino-3-hydroxy-5-methyl-4-isoxazolepropionic acid (AMPA) receptor trafficking and glutamatergic currents through calcium/calmodulin-dependent protein kinase II (CaMKII), promoting tumor-cell depolarization, proliferation, and synapse formation.^22^ In pancreatic adenocarcinoma, tumor-associated Schwann-cell reprogramming promotes progression and invasion through GDNF overexpression and enhanced neurite outgrowth.^23^ Reprogrammed Schwann cells can also form tumor-activated Schwann cell tracks, dynamic migratory structures that may facilitate dissemination.^24^

Structural remodeling becomes immunologically consequential when inflammation alters nerve activity. Cytokine-mediated nociceptor sensitization is a well-established mechanism by which inflammation changes peripheral neural function. In melanoma, tumor-secreted secretory leukocyte protease inhibitor activates neurons, induces calcium influx, and triggers CGRP release while promoting thermal hypersensitivity; within the TME, CGRP directly promotes T-cell exhaustion.^5^ Functional reprogramming of tumor-infiltrating T cells in this setting can also depend on interferon (IFN)-γ signaling and JAK1/2 activation.^25^ Tumors also communicate with nerves through vesicle-mediated routes. Exosomes from patients with head and neck cancer increase neurite outgrowth in PC12 cells in vitro, consistent with enhanced tumor innervation during progression; inhibition of exosome release reverses this effect.^26 27^ These findings reinforce the broader point that tumors actively build, rather than simply occupy, neural niches.

Clinically, these remodeling programs are reflected in aggressive disease behavior. Increased nerve density and altered neural phenotypes correlate with recurrence, invasion, and poor outcome.^28 29^ Perineural invasion (PNI), long viewed mainly as a route of local spread, is better understood as a specialized niche defined by intimate tumor-nerve contact, stromal remodeling, and distinctive immune organization.^30^ Within this niche, malignant, neural, stromal, and immune cells engage in bidirectional communication that supports tumor progression and local immune modulation. PNI also links structural remodeling to immunotherapy response. PNI and cancer-induced nerve injury are associated with impaired responses to anti-programmed death 1 (PD-1) therapy,^6^ indicating that nerve injury is not simply the loss of tumor-promoting innervation but a pathological neural state that can reinforce immune suppression. Tumor cells often align longitudinally along nerve fibers, suggesting that nerves provide both a physical track and molecular guidance cues for directed migration.^30 31^ Neurotrophic signals such as NGF and GDNF, together with chemokine pathways including C-X-C motif chemokine ligand 12 (CXCL12)-C-X-C motif chemokine receptor 4 (CXCR4), promote tumor-cell migration toward and along nerves.^32–36^ The tumor-neural interface therefore has two sides: tumors remodel nerves, and tumors can also acquire neural-like programs themselves.

## Neural-like states in cancer

Once tumors reshape nearby nerves, the boundary between neural and malignant cell states can also blur. Tumors can acquire neural-like features in addition to inducing axonogenesis, neurogenesis, and PNI. These adaptations include neuroendocrine differentiation with production of neuropeptides and classical neurotransmitters,^37–39^ neurite-like projections,^40^ and vesicle-mediated communication pathways.^36^ Because these features are increasingly associated with invasion, metastatic dissemination, and therapy resistance, the central interpretive question is whether they reflect developmental lineage or an inducible tumor state.

### Lineage-derived and neural crest-associated programs

The clearest reference point is provided by tumors whose neural programs reflect developmental lineage. Central nervous system (CNS) tumors, including glioma and ependymoma, are canonical lineage-consistent neural states.^41 42^ These tumors arise from neuroectoderm-derived progenitors such as radial glia and retain neural transcriptional programs, including synaptic machinery, ion channels, and neurosecretory pathways.^43^ In supratentorial ependymomas (ST-EPNs), spatial transcriptomics combined with live-cell imaging revealed marked functional heterogeneity: neuron-like canonical ST-EPN tumor cells, characterized by the fusion of zinc finger translocation associated (ZFTA) with RELA proto-oncogene, NF-κB subunit (RELA), known as the ZFTA-RELA fusion, show saltatory-like migration, defined here as intermittent bursts of rapid, jumping movement rather than axonal saltatory conduction, together with neurite-like extension, retraction, and branching, whereas immature ependymal-like cells remain largely quiescent.^41^ These findings show how CNS-derived tumor cells can co-opt neurodevelopmental migratory programs within the brain microenvironment.

Glioblastoma extends this lineage-derived pattern into a more invasive networked state.^42 44^ Glioblastoma cells can adopt neuron-like and neural progenitor-like transcriptional programs and respond to glutamatergic neuronal input, which drives de novo tumor-microtube formation, coordinated migration, and brain colonization^42 45^ (figure 1).

**Figure 1:**
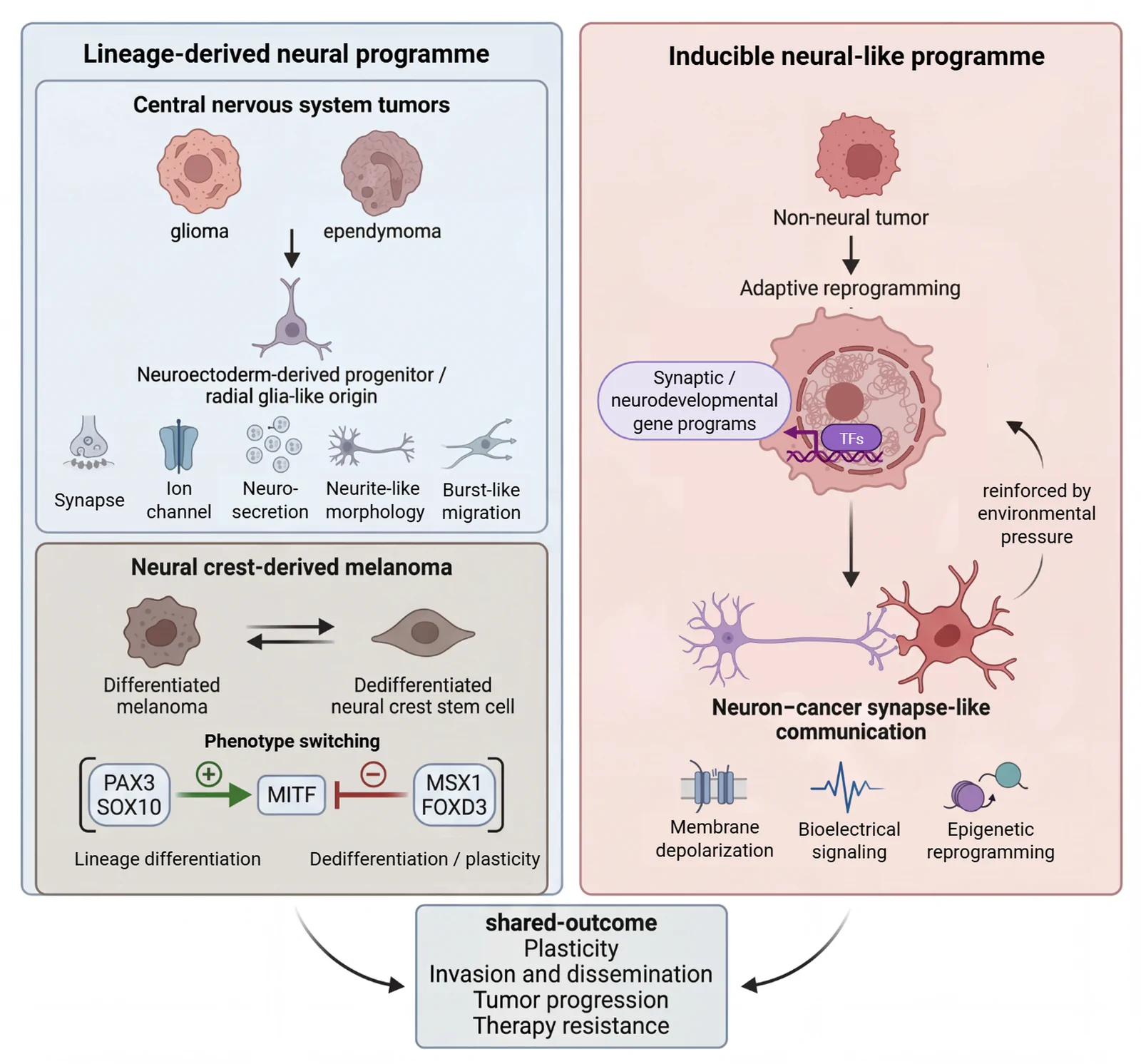
Distinct origins of neural-like programs in cancer. Neural traits in cancer arise through two broad mechanisms: lineage-derived programs and tumor-intrinsic reprogramming. CNS tumors retain neuroectodermal features, whereas neural crest-derived melanoma undergoes reversible phenotype switching governed by the PAX3-SOX10-MITF axis and opposed by MSX1-FOXD3. In CNS tumors, saltatory-like migration refers here to intermittent burst-like cellular movement rather than axonal saltatory conduction. In non-neural tumors, increased chromatin accessibility and activation of transcription factors can initiate adaptive reprogramming. This process drives the activation of synaptic and neurodevelopmental gene programs, enabling tumor cells to acquire neural-like states and engage in synapse-like communication with neurons. Despite their distinct origins, these routes converge on common outcomes, including cellular plasticity, invasion, tumor progression, and therapy resistance. CNS, central nervous system; FOXD3, forkhead box D3; MITF, microphthalmia-associated transcription factor; MSX1, muscle segment homeobox 1; PAX3, paired box 3; SOX10, sex-determining region Y-box 10; TFs, transcription factors.

### Melanoma shifts the argument from retained lineage to reactivated developmental plasticity

Its neural traits are not simply inherited; they are dynamically re-engaged through developmental gene-regulatory networks that control migration, plasticity, and stress adaptation. Within this circuitry, paired box 3 (PAX3) and sex-determining region Y-box 10 (SOX10) cooperate to activate microphthalmia-associated transcription factor (MITF) and maintain melanocytic identity, whereas muscle segment homeobox 1 (MSX1) and factors such as forkhead box D3 (FOXD3) antagonize MITF to promote dedifferentiation.^46^ This balance allows melanoma cells to shift between differentiated and dedifferentiated states, including neural crest stem cell-like phenotypes,^47^ thereby driving phenotypic plasticity and spatial intratumoral heterogeneity^48 49^ (figure 1). These transitions support adaptation to hypoxia, inflammation, and therapy-induced stress.^50 51^ They are also immunologically consequential: SOX10 ablation reduces immune checkpoint molecules, including herpesvirus entry mediator and carcinoembryonic antigen-related cell adhesion molecule 1 (CEACAM1), and restricts tumor growth in a CD8^+^ T cell-dependent manner, linking neural crest-like states to tumor immunogenicity.^52^

### Tumor-intrinsic inducible neural-like programs

Non-neural cancers push this concept further by acquiring neural-like programs despite lacking a neural developmental origin. These inducible states suggest that neural mimicry can emerge as a convergent adaptive response to the TME. Tumors of non-neural origin can acquire neuron-like functional properties, including neuron-cancer synapses, activity-dependent membrane depolarization, and calcium transients, which promote growth and metastatic colonization.^53 54^ These phenotypes are supported by ectopic activation of synaptic and neurodevelopmental gene programs, including axon-guidance and neurotransmission pathways^40^ (figure 1). In small cell lung cancer, this transition is driven in part by Nfib overexpression.^55^ In triple-negative breast cancer, erosion of genome-wide DNA methylation disrupts epithelial lineage fidelity and makes tumor cells permissive to SOX10-driven neural crest-like reprogramming, a state associated with increased plasticity, migration, and poor clinical outcome.^56^ Neural mimicry should therefore be viewed as a recurrent adaptive state rather than as a lineage-restricted property.

## Neural inputs and major ligand-receptor axes in the TME

These lineage-derived and inducible programs converge with a second question: which neural inputs dominate the TME? Neural regulation arises from sensory, autonomic, enteric, and central circuits rather than from a single innervation pattern (figure 2). Sensory neurons detect tumor-associated injury and inflammation and release neuropeptides that act locally on immune, stromal, and malignant cells. Sympathetic, parasympathetic, and enteric circuits regulate immune tone through classical neurotransmitters and neuropeptides, while ascending afferent signals and descending central outputs form a broader tumor-brain-immune axis. The relevant issue is not whether neural input is beneficial or harmful in general, but which circuit state is engaged. This framing reconciles studies in which neural activity supports tumor progression^5^ with studies in which loss of homeostatic input or cancer-induced nerve injury accelerates progression or impairs immunotherapy responses.^6^

**Figure 2:**
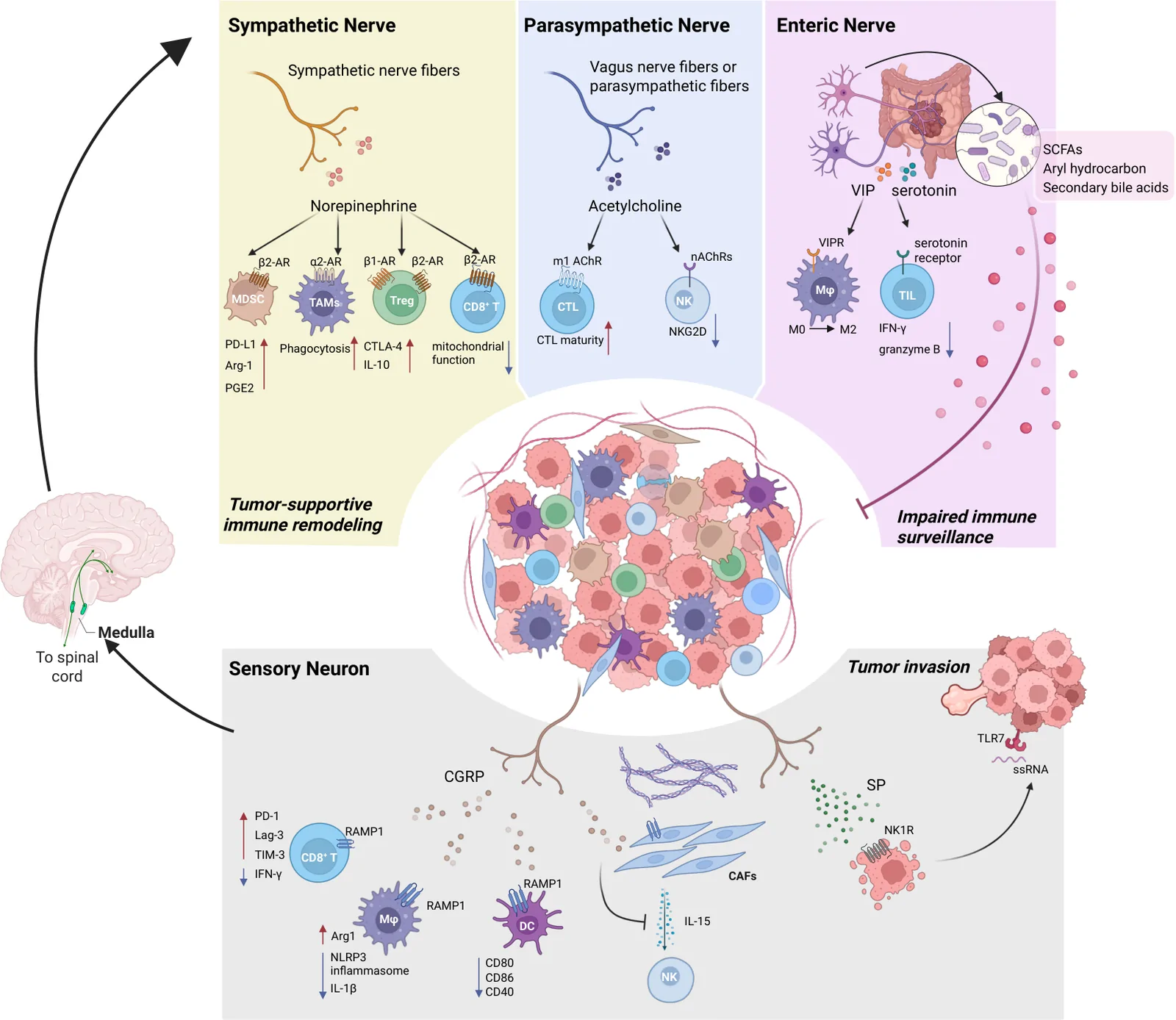
Neural regulation of tumor immunity across sensory and autonomic circuits. Sensory neurons release CGRP and substance P, which can suppress T-cell function, impair antigen presentation, and promote immunosuppressive myeloid states. Sympathetic signaling through β-adrenergic receptors often drives tumor-supportive immune remodeling, whereas parasympathetic acetylcholine signaling modulates T-cell, NK cell, and myeloid function in a receptor-subtype-dependent manner. Enteric neurons and microbiota-derived metabolites further shape immunity through VIP and serotonin signaling. Together, these pathways form an integrated tumor-brain-immune axis whose net effect depends on neural state, receptor landscape, tissue context, and disease stage. Ach, acetylcholine receptor; AR, adrenergic receptor; Arg1, arginase 1; CAFs, cancer-associated fibroblasts; CGRP, calcitonin gene-related peptide; CTL, cytotoxic T lymphocyte; CTLA-4, cytotoxic T-lymphocyte-associated protein 4; DC, dendritic cell; IFN-γ, interferon-γ; IL-1β, interleukin-1β; IL-10, interleukin-10; IL-15, interleukin-15; LAG-3, lymphocyte-activation gene 3; M0, resting macrophage; M2, immunosuppressive macrophage; MDSC, myeloid-derived suppressor cell; Mφ, macrophage; NK, natural killer; NK1R, neurokinin-1 receptor; NLRP3, NOD-, LRR- and pyrin domain-containing protein 3; NKG2D, natural killer group 2D; PD-1, programmed death 1; PD-L1, programmed death-ligand 1; PGE2, prostaglandin E2; RAMP1, receptor activity-modifying protein 1; SCFAs, short-chain fatty acids; ssRNA, single-stranded RNA; TAMs, tumor-associated macrophages; TIM-3, T-cell immunoglobulin and mucin-domain containing 3; TIL, tumor-infiltrating lymphocyte; TLR7, Toll-like receptor 7; Treg, regulatory T cell; VIP, vasoactive intestinal peptide; VIPR, vasoactive intestinal peptide receptor.

### Neuropeptide-regulated tumor immunity

Sensory neuropeptides provide the most local route by which tumor-activated nerves influence immunity. Sensory neurons detect noxious mechanical, thermal, chemical, damage-associated, and microbial stimuli through specialized ion channel receptors. Their afferent fibers innervate barrier tissues such as the skin, lung, and intestine and relay signals to the CNS through cell bodies in the dorsal root, trigeminal, jugular, and nodose ganglia, as well as through enteric ganglia in the myenteric and submucosal plexuses.^57^ These inputs are perceived as pain or itch.^58^ During tumor progression, axonal injury, tissue compression, and tumor-associated inflammation can activate or sensitize transient receptor potential vanilloid 1 (TRPV1)-expressing nociceptors. This activation triggers release of neuropeptides such as CGRP and substance P, which then engage immune and stromal receptors within the TME.^6 59–62^

#### Calcitonin gene-related peptide

CGRP is among the clearest examples of a sensory-neuron output that functions as an immune regulator in cancer. This 37-amino acid neuropeptide signals through a receptor complex composed of calcitonin receptor-like receptor and receptor activity-modifying proteins (RAMPs). RAMP1-3 confer ligand specificity and functional diversity. RAMP1 is broadly expressed in dendritic cells (DCs),^63^ macrophages,^64 65^ and activated T cells,^5 66^ whereas activated CD4^+^ T cells preferentially express RAMP3, which contributes to T helper-cell polarization.^67^ Across multiple tumor types, CGRP levels are elevated relative to adjacent normal tissue, and CGRP and RAMP expression inversely correlate with immune infiltration and patient survival.^38 68–70^

At the immune-cell level, CGRP suppresses antitumor immunity across lymphoid and myeloid compartments. In murine melanoma, enriched and sensitized nociceptors signal through CGRP-RAMP1 to impair CD8^+^ T-cell cytotoxicity and promote exhaustion; CGRP receptor blockade or TRPV1 ablation restores CD8^+^ T-cell function and enhances anti-programmed death-ligand 1 (PD-L1) efficacy, and increased RAMP1^+^ CD8^+^ T cells in human melanoma are associated with worse survival.^5 20^ In head and neck squamous cell carcinoma (HNSCC), sensory denervation, CGRP deficiency, or pathway blockade increases Th1 CD4^+^ T-cell, CD8^+^ T-cell, and natural killer (NK)-cell infiltration, suppresses tumor growth, and increases radiosensitivity.^70 71^ CGRP also restrains macrophage and dendritic-cell inflammatory programs by limiting NOD-, LRR- and pyrin domain-containing protein 3 (NLRP3) inflammasome activation and interleukin (IL)-1β production, enhancing IL-4-induced arginase 1 (Arg1), and impairing antigen presentation.^72^ In medullary thyroid carcinoma, CGRP signaling through a RAMP1-Krüppel-like factor 2 (KLF2) axis suppresses dendritic-cell antigen-presentation and costimulatory programs, including CD80, CD86, and CD40.^73^

The same inhibitory signal can scale from immune-cell state to lymphoid-tissue architecture. Lung adenocarcinoma (LUAD) progression induces CGRP release from nociceptive sensory neurons, which suppresses the differentiation and tumor necrosis factor (TNF)-α production of RAMP1-high macrophages with hematopoietic lymphoid tissue inducer-like activity. By limiting recruitment of C-X-C motif chemokine ligand 13 (CXCL13)^+^ fibroblasts, this pathway blocks tertiary lymphoid structure (TLS) assembly, a feature associated with antitumor immunity and favorable prognosis in LUAD. Consistently, sensory denervation increased TLS number, size, and maturity in LUAD and improved responsiveness to anti-PD-1 therapy.^74^

CGRP can also create immune exclusion through stromal remodeling. In pancreatic ductal adenocarcinoma (PDAC), nociceptor-derived CGRP suppresses cancer-associated fibroblast (CAF)-derived IL-15, limiting NK-cell infiltration and cytotoxicity and promoting progression.^75^ In triple-negative breast cancer, CGRP activates a cyclic adenosine monophosphate (cAMP)–protein kinase A (PKA)–cAMP response element-binding protein (CREB) pathway in fibroblasts, driving their transition toward myofibroblastic CAFs. This transition increases collagen production and deposition, creating a dense stromal barrier that restricts immune infiltration.^76^ CAF-derived NGF can in turn enhance nociceptor CGRP production, establishing a feed-forward stromal-neuronal circuit.^75^

Across these compartments, current evidence points to a canonical CGRP-RAMP-Gαs-cAMP axis, although the downstream circuitry remains incompletely defined. In CD8^+^ T cells, cAMP-PKA signaling inhibits TCR activation,^77^ whereas in macrophages it enhances IL-4-driven signal transducer and activator of transcription 6 (STAT6) phosphorylation.^72^ By contrast, during acute lymphocytic choriomeningitis virus infection, CGRP can promote Th1 responses through RAMP3-CREB signaling.^67^ In cancer, however, tumor-associated CGRP most often behaves as a neural checkpoint: it links sensory-neuron activation to immune exclusion, T-cell exhaustion, impaired antigen presentation, suppressive myeloid polarization, reduced NK-cell infiltration, constrained TLS formation, and therapeutic resistance.

#### Substance P

Substance P offers a more context-dependent sensory-neuropeptide example. It primarily signals through neurokinin-1 receptor (NK1R; encoded by tachykinin receptor 1 [TACR1]) and contributes to dorsal root ganglion-mediated inflammatory pain in cancer,^62 78^ as well as tumor invasion, metastasis, and immune modulation. In murine breast cancer models, NK1R activation selectively induces death of an NK1R-high tumor-cell subset. The resulting single-stranded RNA engages Toll-like receptor 7 (TLR7) on neighboring tumor cells and promotes invasion through a myeloid differentiation primary response 88 (MyD88)-independent pathway^79^; related observations have been reported in PDAC^80^ and HNSCC.^81^ The immune effects of substance P remain less resolved. In a murine model of breast cancer brain metastasis, sustained low-dose substance P reduces tumor-infiltrating myeloid-derived suppressor cells (MDSCs) and synergizes with radiotherapy-induced antigenicity, resulting in regression and reduced metastasis.^82^ Substance P-NK1R signaling may therefore support invasion through tumor-intrinsic programs or enhance antitumor immunity when modulation of suppressive myeloid cells predominates.

### Autonomic nervous system

Sensory neuropeptides explain local nerve-immune signaling, but autonomic and enteric circuits extend neural control to organ-level physiology. The autonomic nervous system comprises the sympathetic nervous system (SNS), parasympathetic nervous system (PNS), and enteric nervous system (ENS). Sympathetic postganglionic fibers coordinate stress responses through catecholamines, including norepinephrine (NE) and epinephrine, acting via α-adrenergic (α1, α2) and β-adrenergic (β1-β3) receptors. Parasympathetic postganglionic fibers mediate rest-and-digest functions through acetylcholine signaling via muscarinic acetylcholine receptors (mAChRs) and nicotinic acetylcholine receptors (nAChRs) and often exert more localized control because of their shorter postganglionic projections.^83^ The ENS, composed of the myenteric and submucosal plexuses, innervates the gastrointestinal tract and releases acetylcholine, nitric oxide, serotonin, CGRP, vasoactive intestinal peptide (VIP), and neuropeptide Y (NPY).^84^ Through its role in intestinal immune homeostasis, the ENS links microbial cues to tumor-associated immune remodeling, particularly in gastrointestinal cancers.^85^

#### SNS-catecholamine axis

Within this autonomic framework, the SNS-catecholamine axis provides the clearest bridge between stress physiology and tumor immunity. Tumors can chronically engage central and peripheral sympathetic pathways through persistent inflammation, pain, metabolic dysregulation, and psychological stress.^86–90^ Surgery-associated tissue injury,^91 92^ chemotherapy-associated and radiotherapy-associated neural or cytotoxic injury,^93 94^ and immunotherapy-associated cytokine release or immune-related adverse events^95^ can further activate the hypothalamic-pituitary-adrenal (HPA) axis and SNS. Sustained catecholamine and glucocorticoid (GC) exposure can then remodel the TME toward an immunosuppressive, pro-tumorigenic state.^96–98^

In immune cells, NE acts largely through β2-adrenergic receptor (β2-AR). In stage I non-small cell lung cancer (NSCLC), β2-AR expression independently predicts poor progression-free survival.^99^ Catecholamine-driven β2-AR activation enhances MDSC survival, expansion, and suppressive function by promoting STAT3 phosphorylation, reinforcing FAS-Fas ligand (FASL) interactions, and upregulating PD-L1 and Arg1.^100^ It also reshapes MDSC metabolism by increasing oxidative phosphorylation and fatty acid oxidation and stimulates prostaglandin E2 (PGE2) production.^101–103^ In CD8^+^ T cells, β2-AR signaling suppresses glucose transporter 1 (GLUT1) expression, reduces glucose uptake and glycolytic flux, and impairs mitochondrial function.^101^ This metabolic disruption compromises effector differentiation and promotes an exhausted tumor-infiltrating lymphocyte (TIL) phenotype.^104–106^ NE also enhances regulatory T-cell suppressive activity through cAMP-dependent induction of cytotoxic T-lymphocyte-associated protein 4 (CTLA-4) and IL-10.^107^ In hepatocellular carcinoma, β1-AR activation promotes mucosal-associated invariant regulatory T cells (Tregs), a population associated with poor prognosis.^108^

In immunocompetent murine models of melanoma, breast cancer, and colon cancer, α2-AR agonists such as clonidine or guanfacine enhance tumor-associated macrophage phagocytosis and restrain tumor growth.^109^ α2-AR activation can also reduce immunosuppression by inducing MDSC apoptosis.^110^ The net effect of catecholamines therefore depends on the AR landscape of the TME: β-adrenergic signaling in MDSCs, Tregs, and CD8^+^ T cells generally favors immune suppression, whereas α2-adrenergic activation can enhance macrophage function and reduce suppressive myeloid populations in selected models.

#### PNS-acetylcholine axis

Cholinergic signaling introduces a receptor-specific counterpoint to catecholamine-driven immune regulation. The mAChR family comprises five subtypes (M1R–M5R), which are broadly expressed across T cells, B cells, and monocytes.^111^ M1R, M3R, and M5R primarily signal through protein kinase C, whereas M2R and M4R inhibit adenylate cyclase.^112^ Accordingly, muscarinic effects in tumors are likely determined by receptor subtype and immune-cell identity. M1R is required for mature effector cytotoxic T-lymphocyte differentiation after TCR engagement and costimulatory signaling in CD8^+^ T cells.^111^ Tumors can also alter muscarinic receptor profiles in immune cells: macrophages from naive mice predominantly express M1R, whereas macrophages from breast tumor-bearing mice upregulate M2R, which enhances PGE2 production and promotes angiogenesis.^113^

Among nicotinic receptors (nAChR) subtypes expressed on immune cells, the predominant form is the homopentameric α7 nAChR, which transduces acetylcholine signals through Ca2^+^-calmodulin-dependent pathways.^114^ IL-12, IL-15, and IL-18 activate NK cells while upregulating α7 nAChR expression. Pharmacologic α7 nAChR activation attenuates nuclear factor-κB expression and nuclear translocation, downregulates NK group 2D (NKG2D), suppresses NK-cell cytotoxicity and proinflammatory cytokine production, and reduces NK-cell-mediated dendritic-cell maturation.^114^ nAChR activation in MDSCs also promotes IL-10 production and strengthens suppressive function.^115^

In orthotopic murine PDAC, bilateral subdiaphragmatic vagotomy increases intratumoral CD8^+^ T-cell infiltration, elevates the Th1/Th2 ratio, and prolongs survival.^116^ Other studies, however, report that loss of vagal tone increases intratumoral TNF-α, expands tumor-associated macrophages, and reduces survival.^117^ These findings point to context-specific effects shaped by tumor site, inflammatory state, and the balance of cholinergic receptor programs.

#### ENS and gut-brain axis

The ENS closes the local-circuit section by linking autonomic regulation to microbial and neuropeptide cues in the gastrointestinal tract. It modulates gastrointestinal tumor immunity through locally released neurotransmitters and neuropeptides, including VIP and serotonin. In murine PDAC and colorectal cancer models, VIP signaling promotes T-cell exhaustion^118^ and skews macrophages toward a pro-tumorigenic M2 phenotype.^119^ VIP antagonism enhances intratumoral T-cell activation, reinforces M1-like macrophage polarization and phagocytic capacity, and synergizes with PD-1 blockade. Serotonin can also promote tumor progression by downregulating IFN-γ and granzyme B production in tumor-infiltrating CD8^+^ T cells.^120^

Microbial abundance and metabolites, including short-chain fatty acids (SCFAs) and neurotransmitters such as serotonin, also influence ENS phenotype and function during development and adulthood.^121^ Conversely, ENS-derived acetylcholine, catecholamines, neuropeptides, serotonin, histamine, and neurotrophic factors influence the composition and structure of the gut microbial community.^122 123^ The gut microbiota and its metabolites can directly enhance cancer stemness and invasion in gastrointestinal malignancies^124–126^ and can promote immune tolerance by inducing regulatory T cells and MDSCs or suppressing cytotoxic lymphocyte activity.^127–131^

The gut-brain axis also provides a transition from local tumor immunity to systemic neuroimmune tone. SCFAs, secondary bile acids, and tryptophan metabolites can reach the CNS and modulate microglial, astrocytic, and neuronal activity, thereby shaping neuroinflammatory tone and neural-circuit function.^132^ SCFAs are key regulators of microglial maturation and activation, underscoring a microbiota-immune-brain axis.^133^ Through central integration, microbial cues can engage neuroendocrine and autonomic outputs, including the HPA axis, and thereby influence systemic immune homeostasis.

### Neuroendocrine control of antitumor immunity

The gut-brain axis leads naturally from local innervation to systemic control. Cancer-neural communication operates not only through nerves within or near tumors, but also through anatomically wired circuits and circulating neuroendocrine mediators. The CNS shapes the TME by modulating sympathetic and parasympathetic efferents that innervate tumors and lymphoid organs and by coordinating neuroendocrine outputs.^134–136^ Conversely, tumors can activate peripheral sensory neurons and transmit afferent signals to the CNS, where they are integrated into broader neuroimmune adaptations.^137^ This architecture links peripheral sensory detection, central autonomic and endocrine integration, and organism-wide immune remodeling.

Recent circuit-mapping studies make this hierarchy explicit. Thus, tumor-derived signals can be detected by nociceptive and vagal afferents, relayed to brainstem and hypothalamic nuclei, and converted into autonomic outputs to peripheral tissues. In lung adenocarcinoma, NPY2R^+^/TRPV^+^ vagal sensory afferents relay tumor-derived signals to vesicular glutamate transporter 2 (VGluT2)^+^ premotor neurons in the rostral ventrolateral medulla, a key sympathetic control center. Circuit activation increases sympathetic efferent tone to the TME, where β2-AR signaling in alveolar macrophages suppresses antitumor immunity.^7^ This example demonstrates that local tumor-derived cues can be transformed into long-range autonomic programs that reshape immune function.^7^

Furthermore, stress-related endocrine pathways provide a clinically pervasive systemic route. Chronic inflammation, metabolic dysregulation, pain, and psychological distress can sustain HPA-axis and SNS activation, altering GC and catecholamine levels. Psychological distress is common in patients with cancer and is associated with higher rates of depression and anxiety than in the general healthy population.^138^ These states can compromise therapeutic efficacy by promoting systemic immunosuppression, metabolic reprogramming, and sickness-associated behavioral changes.^139 140^ Focusing on GCs, elevated cortisol in patients with depression or anxiety has been associated with poorer responses to immune checkpoint inhibitors (ICIs).^141 142^ In mice, chronic social-defeat stress increases GCs and accelerates NSCLC and colorectal cancer by inducing TSC22 domain family protein 3 (TSC22D3), which impairs type I IFN responses in tumor-infiltrating DCs and restricts T-cell activation.^143^ In patients with NSCLC, concomitant corticosteroid treatment is associated with reduced baseline circulating CX3CR1^+^CD8^+^ T cells and diminished ICI responsiveness.^144^ GCs can also promote neutrophil extracellular trap formation, enabling capture of circulating tumor cells and metastatic spread.^145^ Notably, durable recurrence-free survival after resection of a primary metastatic lesion in mice required the TLR9 immunostimulant class C cytosine-phosphate-guanine (CpG) oligodeoxynucleotide (CpG-C) plus blockade of both GC receptor and β-AR signaling.^146^

It is also worth noting that the suprachiasmatic nucleus (SCN) acts as the central pacemaker and synchronizes peripheral clocks across tissues. Through autonomic pathways, the SCN regulates rhythmic immune-cell trafficking to lymph nodes, while cortisol and epinephrine impose opposing circadian control over distinct T-cell subsets.^147^ Patients with locally advanced or metastatic cancer commonly show neuroendocrine circadian disruption, including an earlier but attenuated nocturnal melatonin surge and reduced diurnal amplitude of cortisol secretion.^148^ These disturbances are likely immunologically relevant because immune, stromal, and tumor cells contain peripheral clocks that regulate leukocyte trafficking,^149 150^ cytokine production,^151^ antigen presentation,^152^ cellular metabolism,^153^ and effector function.^154^ Circadian disruption has also been linked to tumor initiation, metastasis, and therapy resistance through effects on cell-cycle regulation,^155^ DNA repair,^156^ metabolism,^157^ and cancer stemness.^158 159^ In breast cancer and melanoma, it can impair antitumor immunity by increasing tumor-associated macrophages and Treg) cells, reducing CD8^+^ T-cell infiltration and activity, and promoting anti-inflammatory macrophage polarization.^160 161^

Together, local circuit mapping, stress endocrinology, and circadian biology extend cancer neuroscience from a nerve-tumor model to a multilevel regulatory architecture. Tumors can activate afferent neural pathways and systemic stress programs that recalibrate sympathetic, parasympathetic, HPA-axis, and circadian outputs. These outputs coordinate immune-cell trafficking, myeloid polarization, T-cell dysfunction, metastatic competence, and therapeutic responsiveness across the TME, lymphoid organs, and distant tissues. Antitumor immunity is therefore shaped by both wired innervation and circulating neuroendocrine signals that broadcast cancer-associated stress states throughout the organism.

## Neural signaling and immunotherapy responses

The mechanistic arc now converges on therapy: do neuroimmune circuits determine who responds, who resists, and who develops toxicity? Cancer immunotherapy has reshaped oncology, but durable benefit remains limited to a subset of patients and acquired resistance is common.^162–165^ Neural signaling can influence these outcomes by regulating immune-cell recruitment, antigen presentation, effector function, and tissue toxicity through neurotransmitters, neuropeptides, and direct axonal interactions with tumor, stromal, and immune cells.^5 166–168^ This creates two clinically relevant questions: whether neural circuits limit antitumor efficacy, and whether they amplify treatment-related adverse events.

Resistance can emerge spatially, where nerve-rich niches shelter suppressive programs from systemic therapy. As tumors become more innervated, intratumoral nerve fibers can provide localized sources of immunomodulatory mediators,^4^ generating pockets of suppression that may persist despite systemic therapy.^6 69^ PNI and cancer-induced nerve injury are associated with poor anti-PD-1 responses, suggesting that neural remodeling can mark or actively drive resistant immune states.^6^ Conversely, disrupting suppressive neural pathways can restore sensitivity: CGRP receptor blockade or TRPV1^+^ sensory-neuron ablation improves CD8^+^ T-cell function and anti-PD-L1 efficacy in melanoma,^5^ CGRP-pathway disruption increases immune infiltration and radiosensitivity in HNSCC,^70 71^ and VIP antagonism enhances intratumoral T-cell activity and synergizes with PD-1 blockade in PDAC and colorectal cancer models.^118^ These examples identify neural signals as actionable regulators of therapeutic response rather than background features of tumor biology.

### Neural regulation of immune checkpoint blockade responses

For immune checkpoint blockade, sympathetic signaling is the best-studied link between stress physiology and resistance. Stress-associated catecholamines, particularly NE and epinephrine, signal through β-ARs expressed by dendritic cells, macrophages, lymphocytes, and many tumor cells.^169–171^ This pathway generally suppresses antitumor immunity by promoting regulatory immune states and impairing cytotoxic lymphocyte activity.^169 172^ Chronic catecholamine exposure can also drive CD8^+^ T-cell exhaustion through β1-AR signaling, which is upregulated in exhausted T cells and restricts proliferation and cytokine production.^169^ Sympathetic inputs can therefore constrain antitumor immunity upstream of checkpoint inhibition.

Nerve injury provides a second, mechanistically distinct route to checkpoint resistance. PNI and cancer-induced nerve injury have been associated with poor anti-PD-1 responses across several tumor types.^6^ In this state, tumor-mediated nerve damage activates neuronal IL-6 and type I IFN programs that promote a chronically suppressive TME and reinforce T-cell dysfunction.^6^ Preclinical studies indicate that this resistance is reversible: denervation, inhibition of neuronal injury signaling, or combined blockade of PD-1 and IL-6 receptor signaling can restore sensitivity.^6^ Neural injury should therefore be interpreted not as a bystander feature of advanced disease, but as an active immune-evasion state. This model has direct implications for ICIs. Anti-PD-1/PD-L1 and anti-CTLA-4 therapies release inhibitory constraints on T cells, but their efficacy depends on sufficiently primed, metabolically competent, and responsive effector cells. If adrenergic or injury-associated signaling has already compromised these upstream requirements, checkpoint blockade alone may not restore effective immunity. Neural signaling may therefore lower the ceiling of ICI responsiveness and contribute to both primary and acquired resistance.

Because these pathways converge upstream of T-cell reinvigoration, β-adrenergic blockade has a strong mechanistic rationale, even though clinical evidence remains incomplete. Preclinically, β-adrenergic blockade reduces T-cell exhaustion, enhances CD8^+^ T-cell function, and improves checkpoint responses in multiple models.^169^ Propranolol also suppresses tumor growth and metastasis while promoting Th1-polarized cytotoxic CD4^+^ T-cell responses and enhancing anti-CTLA-4 activity.^172^ Translational observations are consistent but not definitive: chronic psychological stress has been associated with adverse cancer outcomes, stress-management interventions may reduce cancer risk or slow progression in selected settings,^173 174^ and β-blocker use has been associated with improved outcomes in breast, ovarian, pancreatic, and prostate cancer.^175–178^

In metastatic melanoma, a phase I dose-escalation study found that propranolol plus pembrolizumab was safe and well tolerated, with no dose-limiting toxicities, a recommended phase II dose of 30 mg two times a day, and encouraging preliminary activity, including a 78% objective response rate.^179^ Retrospective studies have also suggested improved outcomes with nonselective β-blockers during immunotherapy.^180 181^ However, a meta-analysis of 11 studies involving 10 156 patients found no significant association between β-blocker use and improved overall or progression-free survival in solid tumors treated with ICIs.^181^ Studies in pancreatic adenocarcinoma, urothelial carcinoma, soft-tissue sarcoma, and esophageal or gastroesophageal junction adenocarcinoma have likewise not shown clear benefit from combining β-blockers with ICIs^182^ (ClinicalTrials.gov identifiers: NCT05451043; NCT04848519; NCT05961761; NCT05651594). Benefit is therefore likely to depend on tumor type, timing, drug selection, neural state, and patient stratification. Future trials should incorporate immune profiling, spatial transcriptomics, and standardized treatment parameters to determine whether specific neural signatures predict response to neuromodulatory combinations.

### Neural influences on adoptive cell therapy and CAR-T/TIL approaches.

Adoptive cell therapy (ACT) extends the same logic from endogenous T-cell reinvigoration to transferred lymphocyte function. ACT, including TIL therapy and chimeric antigen receptor T-cell (CAR-T) therapy, has shown substantial efficacy in selected malignancies, but activity in solid tumors remains limited.^183^ Resistance reflects poor trafficking into tumor tissue, limited persistence in the TME, metabolic stress, and progressive T-cell dysfunction. Neural signaling intersects with each barrier. By regulating cytokine and chemokine production by tumor and stromal cells, it may alter the gradients that direct T-cell migration.^166^ Adrenergic signaling has been implicated in regulation of C-X-C motif chemokine ligand 9 (CXCL9), C-X-C motif chemokine ligand 10 (CXCL10), and C-C motif chemokine ligand 5 (CCL5), all of which are relevant to lymphocyte recruitment.^166 184–186^ Sympathetic signaling may also influence vascular tone and endothelial activation, affecting lymphocyte extravasation into tumor tissue.^171^ Neural circuits could therefore shape both the number and the spatial distribution of transferred cells within tumors. After entry, transferred lymphocytes must persist and remain metabolically fit. Nerve-rich microenvironments may create localized suppressive niches in which transferred T cells rapidly lose effector capacity.^187^ This is relevant because both CAR-T and TIL therapies require durable mitochondrial function and appropriate metabolic programming for persistence and activity.^187 188^ Adrenergic signaling interferes with the metabolic reprogramming required for T-cell activation, including glycolysis and mitochondrial function.^104^ In nutrient-deprived solid tumors, these effects could further compromise transferred lymphocyte function and accelerate exhaustion.^189 190^

These trafficking and metabolic pressures are compounded by broader immunoregulatory effects. Neural pathways may indirectly promote exhaustion by expanding regulatory immune populations and suppressing inflammatory cytokines,^191^ and may also act more directly by altering transcriptional programs associated with dysfunctional T-cell states.^71 166 187^ Direct clinical evidence remains limited, but the mechanistic logic supports the hypothesis that neural signaling contributes to the limited durability of ACT in solid tumors. Practical strategies follow from this hypothesis: combine ACT with neuromodulatory agents such as β-blockers, target nerve-associated immunosuppressive niches directly, or engineer transferred lymphocytes to resist neural suppression. Integrating neural signaling features into ACT datasets and spatially mapping neuroimmune interactions in human tumors should help identify the patients and tumor states most likely to benefit.

### Neuroimmune contributions to immunotherapy-related adverse events

The same circuitry that shapes efficacy may also shape toxicity. Cancer immunotherapy-associated toxicities include ICI-related immune-related adverse events (irAEs) and immune effector cell-associated toxicities after CAR T-cell therapy. ICI-associated irAEs commonly involve barrier and endocrine tissues, including skin rash or pruritus, colitis or diarrhea, thyroid dysfunction, and hepatitis.^192^ Neurological irAEs are less common, occurring in approximately 1–5% of patients,^193 194^ although incidence increases with combined PD-1 and CTLA-4 blockade and has been reported to reach 12% in a systematic review.^195^ By contrast, neurotoxicity is a defining complication of CAR T-cell therapy: immune effector cell-associated neurotoxicity syndrome (ICANS) develops in approximately 26.9% of treated patients, with severe or high-grade neurotoxicity in approximately 10.5%.^196^

Pruritus illustrates how neural mechanisms can convert immune activation into persistent symptoms. Persistent pruritus is a common cutaneous irAE, affecting up to 37% of patients treated with anti-PD-1 antibodies, and is often refractory to antihistamines. In mouse models, repeated nivolumab directly activates MrgprA3^+^ pruriceptors and induces IL-2 receptor β and γ subunit expression in these neurons. This amplifies IL-2 signaling through a neuroimmune positive-feedback loop that sustains chronic itch.^197^ Thus, neural pathways can act as amplifiers, not only sensors, of immunotherapy-induced toxicity. Whether sensory or autonomic circuits contribute to other ICI-related irAEs, cytokine release syndrome, or ICANS remains largely unexplored. Because many irAEs arise in densely innervated barrier organs and present with sensory or autonomic symptoms, neuroimmune circuits may offer steroid-sparing targets that mitigate toxicity while preserving antitumor immunity.

## Distance-dependent neuroimmune signaling

Mechanistic claims become actionable only when they are anchored in tissue architecture. Spatial omics links mechanism to anatomy by showing where tumor-neural-immune interactions occur within intact tissue. This is especially important in cancer neuroscience because nerves are filamentous, sparsely distributed, and organized across multiple length scales. Spatial proteomic and transcriptomic platforms can distinguish neuronal, glial, immune, stromal, and malignant compartments and reveal how their physical organization influences tumor behavior, immune niches, and therapeutic vulnerabilities.

### Spatial proteomics

PDAC provides an entry point for spatial proteomics because it is highly aggressive, heavily innervated, and characterized by frequent PNI. Using high-plex spatial proteomics, Lyman *et al* examined early pancreatic intraepithelial neoplasia (PanIN) lesions and found neurons and glial cells closely associated with mixed M1/M2-polarized macrophages and T cells, revealing structured neuroimmune niches.^198^ They also identified TLSs adjacent to PanIN lesions that appeared more immature than TLSs near the PDAC border, suggesting stage-specific immune organization. These spatial protein patterns reveal neuronal and immune heterogeneity that transcriptomic analyses alone might miss and support a role for early neuroimmune organization in PDAC progression.

The same approach shows that neuroimmune architecture varies across tumors and patients. In prostate cancer, multiplex imaging of localized tumors showed that androgen receptor-positive regions display disrupted spatial organization, heterogeneous clustering, and frequent proximity to stromal, immune, and neuronal elements, indicating extensive TME remodeling; these analyses also revealed patient-specific spatial architectures even among tumors with similar pathologic grade.^199^ Complementing this work, Xia *et al* used the Akoya PhenoCycler-Fusion platform with tissue clearing to visualize mouse prostate tissue in three dimensions, enabling high-resolution mapping of neuronal networks and neuroepithelial interactions that are difficult to capture with conventional approaches.^200^

Brain metastasis and breast cancer studies extend this spatial view to distinct neuroimmune niches. Using GeoMx Digital Spatial Profiling, Mendoza-Valderrey *et al* mapped neuronal, immune, and tumor proteins in melanoma-derived and non-melanoma-derived brain metastases.^201^ Melanoma metastases contained dense neuronal clusters colocalized with T cells and macrophages, whereas non-melanoma metastases were less immunogenic and showed weaker neuroimmune interactions, emphasizing that neuroimmune niche architecture can be tumor-type specific. Similarly, Bjørnstad *et al* applied imaging mass cytometry with a 38-plex antibody panel to breast cancer samples and found doublecortin-expressing neural progenitor cells within the tumor stroma, where they were spatially associated with higher tumor grade and poorer survival.^202^ In vitro, crosstalk between breast cancer cells and neural progenitors induced proteomic changes in both cell types, particularly in axon-guidance and nervous system development pathways. These findings support a role for neural progenitor cells in shaping the breast cancer microenvironment.

Together, these studies reframe nerves as organized components of the tumor ecosystem rather than incidental structures. The strength of spatial proteomics lies in mapping cell state and protein expression in situ, revealing neural, immune, and tumor-cell relationships that are obscured in bulk assays. Its limitations are equally important: detection depends on antibody availability and specificity, rare proteins and subtle cell states may be missed, and even high-plex platforms can lack the resolution needed to resolve subcellular structures such as synapses. Spatial proteomics also creates analytical challenges. High-dimensional datasets require robust segmentation, denoising, normalization, and integration workflows, and limited standardization can hinder cross-study comparison. Most platforms also remain fundamentally two-dimensional, capturing a single tissue plane and potentially missing the three-dimensional architecture and branching patterns of nerve fibers.^203–205^ Despite these constraints, spatial proteomics is already exposing neural circuits of tumor progression and revealing therapeutic vulnerabilities that are difficult to infer from bulk tissue profiling.

### Spatial transcriptomics

Spatial transcriptomics adds the gene-expression layer to this anatomic map. This advantage is particularly relevant for nerves, which are filamentous and unevenly distributed within tumors. By preserving microanatomic relationships, spatial transcriptomics can assess interactions across distances, from direct cell contact to paracrine signaling. It thereby supports ligand-receptor analysis, immune-state mapping, and neighborhood-based frameworks for biomarker discovery.

In PDAC, spatial transcriptomics makes nerve geometry analytically useful. Chen *et al* integrated single-nucleus and single-cell sequencing with 10x Genomics Visium V2 spatial transcriptomics to map PNI, its effects on surrounding cell types, and PNI-associated niches.^167^ Their study demonstrates a major strength of spatial transcriptomics: using nerve geometry as an analytical scaffold. By applying a distance-based framework, they modeled near-contact and paracrine effects and treated nerve-influenced cells as coordinated neighborhoods rather than isolated cells.^206^

The same framework can prioritize molecular targets for validation. Lakis *et al* used the Bruker GeoMx Digital Spatial Profiler with RNA probes to profile cancer and nerve compartments in PNI and non-PNI foci.^207^ They identified PNI-associated changes in immune programs, nerve-injury pathways, and axon-guidance pathways and used ligand-receptor analysis to predict direct cancer-nerve interactions.^207^

Applications beyond PDAC show how spatial transcriptomics bridges discovery and validation. In oral cancer, GeoMx digital spatial profiling (DSP) profiling of nerves near and far from tumors identified a dual program of axonogenesis and stress response in tumor-proximal nerves, supported by dual S100 and cytokeratin immunohistochemistry.^208^ In glioblastoma, 10x Genomics Visium spatially validated an inverse relationship between neurosynaptic features and immune regulation identified by single-cell RNA sequencing,^209^ while spatial colocalization analyses defined distinct interaction clusters.^195^ By linking spatial gene expression to histology and perturbation-ready hypotheses, these studies position spatial transcriptomics as a bridge between single-cell sequencing, histology, and functional experimentation.

The analytical ecosystem is expanding quickly enough to make these questions increasingly tractable. Sequencing-based platforms such as Visium, Slide-seq, and GeoMx DSP and imaging-based platforms such as CosMx spatial molecular imaging and Xenium are moving toward broader transcriptome coverage and finer resolution. Computational tools, including Seurat,^210^ CellChat,^211^ Scanpy,^212^ Giotto,^213^ and Squidpy,^214^ support clustering, annotation, cell-cell interaction analysis, spatial localization, and multimodal workflows tailored to specific biological questions. Important limitations remain. Multimodal integration is still difficult, and robust methods for aligning and jointly analyzing multiple spatial datasets are evolving. Neuronal cell bodies and nuclei often lie outside tumor regions, restricting analysis of nerve-cancer interactions to axonal transcription measured in situ. Whole-transcriptome spatial transcriptomics is also largely descriptive, so functional perturbation is needed to establish causality. Nevertheless, its descriptive breadth is valuable: spatial transcriptomics preserves context for neural signaling gradients, neuroimmune-tumor colocalization, and nerve-influenced niches. Targeted panels can then refine these questions while maintaining spatial resolution.

In this sense, spatial omics supplies the anatomic layer needed to translate cancer neuroscience. It connects molecular pathways to measurable tissue biomarkers that can inform patient stratification, target selection, and clinical decision-making. Most importantly, it can distinguish neighborhoods in which neural inputs appear homeostatic, tumor activated, or tumor injured, providing a basis for rational neuro-targeted therapies rather than indiscriminate modulation of neural signaling.

## Conclusion and future perspectives

The central argument of cancer neuroscience is moving from description to mechanism. Tumors remodel innervation, engage peripheral and central circuits, and use neuropeptide, neurotransmitter, neurotrophic, and neuroendocrine pathways to shape malignant growth and immunity. Tumor and stromal cells can also acquire neural-like states that expand the repertoire of tumor-neural communication. These processes converge on a multidirectional tumor-neuroimmune interface that integrates structural remodeling, cell-state plasticity, ligand-receptor signaling, and systemic physiology.^215^

Sensory, sympathetic, parasympathetic, enteric, and systemic neuroendocrine pathways each regulate immune-cell function, but their effects vary with tumor type, anatomic site, receptor repertoire, disease stage, and treatment context. Homeostatic circuits may support tissue integrity and immune surveillance, whereas tumor-activated or tumor-injured programs can drive immune evasion and therapeutic resistance. Making this principle testable will require cellular, spatial, and circuit-level resolution. Key priorities include defining the neural subsets and receptor programs that dominate in each tumor context, separating lineage-derived neural identity from inducible neural mimicry, and resolving how distance-dependent neuroimmune signaling is organized in situ. Integrating spatial omics with functional perturbation, in vivo circuit interrogation, and systemic neuroendocrine profiling offers a route to connect anatomy with immune regulation and therapeutic vulnerability.

The therapeutic implication is that neural circuits may shape responses to conventional therapies, ICIs, ACT, and treatment-related toxicities. Neural interventions could therefore reprogram the TME, improve immunotherapy, and reduce adverse events, but only if they are matched to the relevant neural state: preserving homeostatic circuits, blocking tumor-activated signaling, or interrupting pathological nerve-injury programs. Neuroimmune crosstalk is therefore a fundamental dimension of cancer biology. A mechanistically grounded understanding of this interface should reshape models of tumor ecosystems and enable the development of neuro-informed immunotherapeutic strategies.
